# On the design of deep learning-based control algorithms for visually guided UAVs engaged in power tower inspection tasks

**DOI:** 10.3389/frobt.2024.1378149

**Published:** 2024-04-26

**Authors:** Guillaume Maitre, Dimitri Martinot, Elio Tuci

**Affiliations:** ^1^ Faculty of Computer Science, University of Namur, Namur, Belgium; ^2^ Qualitics SRL, Ans, Belgium

**Keywords:** CNNs, U-net, synthetic imagery, autonomous uavs, photogrammetry, control system

## Abstract

This paper focuses on the design of Convolution Neural Networks to visually guide an autonomous Unmanned Aerial Vehicle required to inspect power towers. The network is required to precisely segment images taken by a camera mounted on a UAV in order to allow a motion module to generate collision-free and inspection-relevant manoeuvres of the UAV along different types of towers. The images segmentation process is particularly challenging not only because of the different structures of the towers but also because of the enormous variability of the background, which can vary from the uniform blue of the sky to the multi-colour complexity of a rural, forest, or urban area. To be able to train networks that are robust enough to deal with the task variability, without incurring into a labour-intensive and costly annotation process of physical-world images, we have carried out a comparative study in which we evaluate the performances of networks trained either with synthetic images (i.e., the synthetic dataset), physical-world images (i.e., the physical-world dataset), or a combination of these two types of images (i.e., the hybrid dataset). The network used is an attention-based U-NET. The synthetic images are created using photogrammetry, to accurately model power towers, and simulated environments modelling a UAV during inspection of different power towers in different settings. Our findings reveal that the network trained on the hybrid dataset outperforms the networks trained with the synthetic and the physical-world image datasets. Most notably, the networks trained with the hybrid dataset demonstrates a superior performance on multiples evaluation metrics related to the image-segmentation task. This suggests that, the combination of synthetic and physical-world images represents the best trade-off to minimise the costs related to capturing and annotating physical-world images, and to maximise the task performances. Moreover, the results of our study demonstrate the potential of photogrammetry in creating effective training datasets to design networks to automate the precise movement of visually-guided UAVs.

## 1 Introduction

The development of Unmanned Aerial Vehicles (UAVs) and their large scale commercialisation offer new solutions to those tasks that pose significant risks to humans (Hallermann and Morgenthal, 2013; Reagan et al., 2018; Albani et al., 2019). One of these tasks is the inspection of power towers, traditionally carried out by humans climbing the tower’s reticular structure to great heights with special inspection equipment. This task makes technicians subject to risks of falls, and to injuries caused by contact with the high-voltage components. UAVs can be used to avoid to expose personnel to these risks by directly replacing humans in the inspection of the towers as well as of the electrical components (Baik and Valenzuela, 2019; Maître et al., 2022).

Beyond safety, the cost-effectiveness of UAVs deployment is a significant advantage because UAVs reduce the direct and indirect costs associated with maintenance operations of the electricity transmission network. For example, inspection-by-human methods might necessitate temporary shutdowns of the power system. The shutdown, while reducing the risks of injury, it increases the disruption to customers. UAVs can instead operate with minimal or no significant impact on power transmission and distribution to customers (e.g., no preventive shutdown). Moreover, UAVs enable frequent and thorough inspections without the same level of resource investment, leading to a more regular maintenance cycle and an earlier detection of potential issues, ultimately extending the lifespan of infrastructure and ensuring consistent power delivery to consumers.

In terms of operational efficiency, UAVs offer unprecedented speed and convenience. A UAV can potentially inspect a target area faster than what it would take a ground crew to inspect it “manually”. This efficiency translates into quicker turnaround time for diagnostics, allowing for more agile responses to emerging issues. Additionally, the ability to inspect multiple towers in a single flight session streamlines the maintenance process, further driving down operational costs. If equipped with high-resolution sensors, UAVs can capture detailed imagery and data, which, when analysed with dedicated algorithms, can lead to rapid diagnostics of structural integrity and to the evaluation of the risks of failure of the high-voltage components. This can reduce the likelihood of occurrence of error in data collection and analysis.

Nowadays, the companies that make use of UAVs for power tower inspection, tend to teleoperate the unmanned aerial vehicles. This requires that the vehicle remains within the human-controller’s view which defines the desired flying trajectories of the vehicle around the tower while avoiding collisions. When the UAVs is not fully teleoperated, it is “programmed” to execute a predefined path plan with a waypoint system, where the UAV is first guided to the designated power tower, and then it moves along the designed trajectory (De Filippis et al., 2012; Ergezer and Leblebicioglu, 2013; Roberge et al., 2013; Lin and Saripalli, 2017; Aggarwal and Kumar, 2020). During the inspection, the UAV may use proximity sensors such as LiDAR, sonar, and radar to assess the environment and to avoid obstacles (Jordan et al., 2018). Path planning might also integrate the drone’s camera as a sensor to detect specific objects requiring attention or avoidance. Whether carried out by teleoperated UAVs or by UAVs programmed to execute a specific path around the tower, the inspection is generally divided into two distinctive phase: the data collection, which happens while the UAVs flies around the structure, and the data analysis, which is carried out “offline” after all data has been collected.

We believe that, the UAVs technology could be exploited in an alternative and potentially more efficient way by providing UAVs the means to autonomously navigate (instead of being teleoperated or programmed to move) around the power tower. A first advantage is that, an autonomous UAVs can develop more informative (from the inspection perspective) flying trajectories, since it can directly control its movements using the readings of multiple sensors directly mounted of vehicle, which provide a perspective on the structure that is precluded to a human controller based on the ground. Moreover, an autonomous UAVs, if equipped with the required technology, can integrate the data collection and the data analysis phase into a single process in which the UAVs movements are determined by the result of the “online” data analysis. This can improve the system efficiency by allocating time and resources only to those parts of the tower that are in needs of in-depth inspection.

This paper aims to contribute to the development of the technology to automate the inspection task of power towers by UAVs, by designing and evaluating methods to exploit camera-based technologies to automate the detection of the tower reticular structure. In the next Section, we illustrate how and why we think this technological innovation can be achieved by exploiting a methodological toolkit made of state-of-the-art solutions already available to researchers.

## 2 Background

The capability to precisely detect and position the tower in a local (i.e., UAV based) framework of reference can be considered a necessary precondition to allow UAVs to develop obstacle-free and potentially informative trajectories as mentioned above. Visually-guided UAVs equipped with state-of-the-art image segmentation algorithms, such as convolution neural networks (hereafter, CNNs), could position themselves relative to the power tower to maximise the quality of the data collection and data analysis process, while avoiding issues such as overexposure, or blurriness of the image that could interfere with the navigation and data collection task (Maître et al., 2022).

However, designing the technology to provide visually-guided UAVs that perceive the surrounding environment with CNNs-based technology that provides the visual acuity necessary to perform the inspection task poses unique difficulties (see Jordan et al., 2018; Lei et al., 2020). First of all, the use of some of the state-of-the-art algorithms for image segmentation, such as CNNs (Girshick, 2015; Badrinarayanan et al., 2017; Bello et al., 2019; Fan et al., 2021; Qin et al., 2022) demands an embedded computing platform to speed up the image-segmentation process. The computational demands of this type of platforms can adversely affect the UAVs’ flight capabilities since the more substantial the computational load, the more limited the UAVs flight endurance becomes. An effective balance between computational power and flight efficiency is crucial to ensure an UAV completes its inspection task without the need of technical breaks (e.g., to recharge batteries).

Moreover, contrary to other domains such as self-driving cars, where CNNs can be relatively easily trained to detect the road signs (see Shustanov and Yakimov, 2017; Zhang et al., 2020), the lane marking (see Yang et al., 2019), or other elements of urban and non-urban visual scenes (see Maqueda et al., 2018; Ouyang et al., 2020), in the domain of UAVs for power tower inspection task there is an issue with the training of the CNNs-based technology. This is due to multiple factors including: i) the difficulty to train CNNs to detect the reticular structure of a tower without confusing it with other similar elements in the scenes such as branches, antenna, *etc.* (Maître et al., 2022); ii) the difficulties related to the collection of a sufficiently large body of images to train robust CNNs capable of copying with the complexity and variability of conditions in which a UAVs are required to operate; (Johnson and Khoshgoftaar, 2019; Shorten and Khoshgoftaar, 2019); iii) the difficulty to test and to validate new technical solutions and algorithms while avoiding the risks of damaging both the UAVs and the electricity structures, due to the fact that towers are often located in remote and difficult to access locations.

In order to overcome the negative influence of the above mentioned factors, we show that it is possible to design computationally light CNNs that, when trained with a combination of physical-world and synthetic images can be extremely effective in distinguishing power towers from background in a variety of scenarios. The type of CNN we use in this study is referred to U-NET (Ronneberger et al., 2015a), a neural network originally developed for biomedical applications, which excels in tasks requiring precise localisation, such as tumour detection in radiology images (Ronneberger et al., 2015a; Siddique et al., 2021). Beyond healthcare, U-Net has been successfully employed in other tasks including satellite imagery analysis and agricultural monitoring (see Wei et al., 2019; Su et al., 2021; Wagner et al., 2019; Freudenberg et al., 2019; Ivanovsky et al., 2019, for example).

We train U-NETs with the support of synthetic images, generated with a simulator that we designed to be accurate in reproducing the characteristics of the different scenarios in which power towers can be found. The development and the exploitation of synthetic data for the training of neural networks is a practice in use since a long time (Hinterstoisser et al., 2019; Nikolenko, 2021), which concerns both processes to augment or modify physical-world images and processes that create entirely simulated (i.e., non-physical) images, referred to as synthetic images (Gupta et al., 2016; Ros et al., 2016; de Melo et al., 2022). The use of synthetic images facilitates the expansion of datasets with the possibility to control the frequency of appearance of specific features or elements in the scenes used for training and evaluation, in order to reduce the risks of over-fitting (Hinterstoisser et al., 2019; Shorten and Khoshgoftaar, 2019).

We mentioned above that we develop synthetic images with a simulator. Simulation environments are extensively used in many research domains to speed-up and facilitate the design, the test, and the validation process of software and hardware components. Simulators are particularly popular in the domain of robotics, where different techniques have been developed to limit the performance loss while porting solutions from simulation onto the physical hardware, due to inevitable differences between the model and the physical world (see N.Jakobi et al., 1995; Miglino et al., 1995; Scheper and Croon, 2017). In this study, we make use of a combination of specific pieces of software to develop a simulator that generates highly accurate model of power towers to be integrated in a variety of highly realistic simulated scenes. The results of our research work show that the synthetic images generated with our simulator can undoubtedly help to speed up and facilitate the training of robust state-of-the art image segmentation algorithms such as computationally light CNNs. We show that the U-Net trained with the support of synthetic images are capable of distinguishing power towers from the background in physical-world images taken by cameras mounted on UAVs while operating the inspection task in different scenarios. Moreover, the simulator we develop in this research work can also be used in the future to test, and to validate different methodological solutions related to the engineering of autonomous UAVs for the inspection of power towers. By accelerating the data collection process and by minimising the costs, the methodological toolkit illustrated in this study is instrumental in developing an advanced autonomous UAV system for micro-analysis of power towers across various natural environments.

## 3 Methods

In this section, we describe the methods used to train a Convolution Neural Network to autonomously identify in camera images, power towers. The image segmentation process is meant to contribute to automate the inspection of these important structural elements of electricity grids by autonomous drones. In particular, we describe the simulator used to generate synthetic images (see Section 3.1), the image datasets used to train the network (see Section 3.2), and the type of convolution neural network used to segment the images (see Section 3.3).

### 3.1 The simulator

In this study, we use the AirSim simulator Shah et al. (2017) to create scenarios that model both natural settings with power towers, and the drone which operates in these settings to inspect the towers. AirSim is a state-of-the-art digital library tailored for AI research. It is designed to create highly realistic environments for different types of autonomous vehicles, including cars and drones. AirSim employs Unreal Engine 4 (available at https://www.unrealengine.com), a powerful and versatile game engine renowned for its high-quality graphics and complex simulations, that provides support to manage the graphical aspects of a simulation, to create diverse scenarios, and to address physics-related tasks. Additionally, AirSim integrates a drone control system and a weather API, further augmenting the engine’s effectiveness in offering a realistic and reliable simulated environment. As far it concerns drones, AirSim allows to model the flight dynamics as well as to generate images from a camera mounted on the drone.

In our simulated scenarios, power towers are located in the following different types of setting.

**FIGURE 1:**
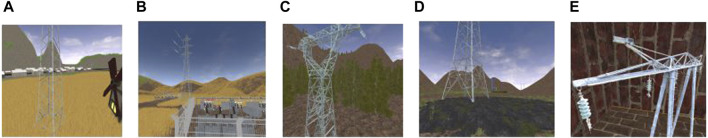
Visualisation of different Photogrammetrically captured power towers integrated into Unreal Engine and AirSim simulator. The image in: **(A)** represents the village setting; **(B)** showcases the cornfield setting with electrical facilities inside it; **(C)** represents the forest and mountainous environment; **(D)** the cobblestone floor and dirt settings; **(E)** represents the high-contrast enclosure setting.

• The Village: This setting introduces the complexity of built-up areas, with structures that could obscure parts of the towers, reflecting the intricacies of human settlements. The village scenario also encompasses a wide variety of colours and contrasts, from the reddish hues of brick walls to the diverse palette of European roof tiling, enhancing the model’s ability to process images with complex colour dynamics (see also Figure 1A).
• The Cornfield: This setting depicts a vast agricultural expanse with height variations posed by crops, representing challenges similar to those found in extensive farmlands. This scenario offers a relatively flat sight line compared to other environments, providing a contrast in visual complexity with power towers that are integrated in uniform and less cluttered backgrounds (see also Figure 1B).
• The Mountain/Forest: This setting is characterised by uneven terrain and dense foliage, mimicking the obstructed views and variable altitudes encountered in natural, undeveloped areas (see also Figure 1C).
• The Cobblestone/Dirt Area: This settings, simpler than the others in structure, depicts an open field with minimal obstructions allowed for straightforward flight paths, akin to those over accessible terrain (see also Figure 1D).
• The High-Contrast Enclosure: The setting refers to a featureless environment, bounded by tall walls, crafted to contrast starkly with the power tower. It creates a unique challenge of visual differentiation in a high-contrast scenario, which is meant to replicate isolated conditions where background distractions are minimised. This environment also helps to generate data for saturated luminosity scenarios (see also Figure 1E).

Each of the above scenarios is chosen for its relevance to the European landscape, reflecting the broad array of settings in which power towers are commonly situated. By integrating the 3D model of power towers within each scenario, at pseudo-randomly chosen positions, we generate a variety of different operating conditions in which the drones is required to carry out the inspection process.

To ensure the simulator provides a reliable models of natural settings, we integrated into the simulated environment Photogrammetrically reconstructed 3D models of power towers (see Figure 1). Photogrammetry is a technique that creates measurements from pictures (Schenk, 2005; Remondino et al., 2012; Goncalves and Henriques, 2015). It is based on the principle that the three-dimensional coordinates of a point on an object can be determined by measurements made in two or more photographic images taken from different positions. The Photogrammetrically reconstructed models accurately reproduce aspects (e.g., the structural as well as textural elements) of physical power towers, by greatly enhancing the realism of our simulator.

The incorporation of photogrammetry-derived models of power towers is meant to improve the effectiveness of synthetic images when used to train convolution neural network for the segmentation process. For example, photogrammetry-based models of towers are characterised by a high-resolution textures and geometrically precise structural details. These properties help to improve the adherence of synthetic scenarios to physical world conditions. Thus, they are instrumental in enabling convolution neural network to recognise intricate features and nuances, which are essential for a precise and accurate image-segmentation process. Another significant advantage of photogrammetry is related to the systematic controlled of some of the potential sources of variability. Synthetic images generated through photogrammetry enable systematic manipulation of various factors, such as lighting conditions, object orientations, and scale. By controlling these elements, we can adopt a more structured and effective approach to training and validating convolution neural networks, ensuring they are well-prepared for a variety of scenarios. Cost-effectiveness is another key benefit. Creating synthetic images via photogrammetry can be less expensive than the labour-intensive and often costly process of collecting physical-world images. This is particularly relevant for scenarios that would otherwise necessitate extensive travel, obtaining various permits, and undertaking time-consuming data collection efforts, especially in challenging or inaccessible locations. Moreover, the use of photogrammetry enhances safety. Generating synthetic images through this method eliminates the need for personnel to be in hazardous environments or close to dangerous structures, such as high-voltage power towers, during the image collection phase. This significantly reduces the risks of accidents and occupational hazards, making it a safer alternative to traditional data collection methods. Finally, by strategically utilising photogrammetry, we enhance the quality and realism of the dataset used for training, with the objective to improve the network ability to deal with the unpredictable physical-world conditions.

### 3.2 The image datasets

Convolution neural networks have been trained to segment images containing power towers using three different datasets: the synthetics dataset, the physical-world dataset, and the hybrid dataset.

The images of the synthetic dataset comprises five structurally different photogrammetrised power towers, placed in different positions of the five scenarios simulated with the AirSim simulator as illustrated in Section 3.1. The images have been generated from a camera mounted on a drone which is manually flown (with the support of the AirSim flight dynamics library) along a predefined trajectory around the towers. The simulated camera model a Zenmuse P1 with 35 mm focal lens locked in forward facing mode. The trajectories of the drone mimics the navigation paths recorded by physical drones while inspecting physical power towers. The use of these flight trajectories in combination with accurate models of the physical towers guarantees that the images reflect the large variability in the operating conditions encountered by physical drone during the inspections and the complexity of the segmentation task. This refers to the fact that images are also taken from “undesired” positions, due to manoeuvres to avoid obstacles (e.g., branches), or being affected by phenomena such as light reflection. The synthetic images are automatically annotated, with each pixel labelled as either part of a tower or of the background.

The physical-world dataset contains images displaying physical power towers under different environmental conditions varying in terms of morphology, colour, textures of the tower, as well as in terms of ambient lighting, background scene. In some of the images, there are also elements (e.g., branches of trees, leaves) that occlude the view of part of the power towers. The cameras used to acquire the images are DJI Zenmuse X5S with 35 mm focal lens equipped on a DJI M210 and a DJI Zenmuse P1 with 35 mm focal lens equipped on a DJI M300. The flight security distance respected was of 5 m from any structural parts of the power tower and the electric cables. The flight were performed by professional pilot during audits and with the agreement from the electricity grid operator.

The hybrid dataset is generated by merging all the physical-world images with all the synthetic images. This dataset serves as a comprehensive training resource, blending the clear, high-quality aspects of synthetic images with the intricate and varying characteristics of physical-world images.

The datasets used for training are made of 1080 images and those for validation are made of 180 images. The statistical properties of the datasets are shown in Table 1. This analysis has been carried out on the resized images used from training and validation. This distribution is calculated around the resized images that will be used during the experiment. For each dataset, the ground truth grayscale images are marked with 0 pixels for non-power tower regions and 255 for power tower pixels.

**TABLE 1:** Table showing statistical properties of the different datasets for training and validation. In particular, it is shown the mean and the median of negative and positive pixels. The table also shows the number of images without any positive pixels. The positive pixels represent the power tower structure while the negative pixels represent the other parts of each image.

Dataset	Mean negative	Mean positive	Median negative	Median positive	w/positive
training *R*	75.2	24.8	75.68	24.32	43
validation *R*	74.66	25.34	75.18	24.82	4
training *S*	81.88	18.12	83.15	16.85	108
validation *S*	81.95	18.05	82.69	17.31	9
training *H*	77.54	22.46	79.13	20.87	72
validation *H*	78.01	21.99	79.09	20.91	3

### 3.3 The convolution neural network

The convolution neural network used in this experiment is an Attention U-Net architecture Ronneberger et al. (2015a), meticulously engineered for real-time power towers image segmentation tasks. We choose an Attention U-Net because it efficiently balances performance with computational pragmatism. For example, this type of network can be trained in a relatively short amount of time and with modest hardware requirements. This makes the U-Net architecture an optimal choice for rapid prototyping, since it allows for quick iteration and deployment in live operational settings, while ensuring reliability and responsiveness to the dynamic demands of drones-based surveillance and analysis.

**FIGURE 2:**
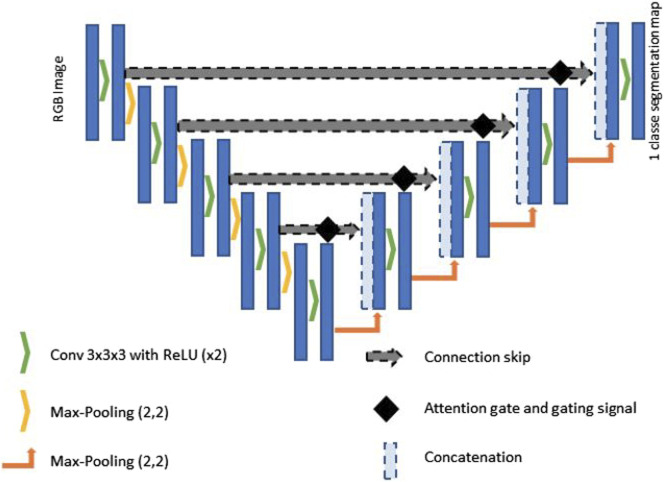
Diagram of the structure of the Attention U-Net Convolutional Neural Network. This structure is adapted from the one illustrated in Oktay et al. (2018), with the addition of an extra convolution and deconvolution layer.

Central to the architecture is an encoder-decoder structure, augmented by skip connections (see Figure 2). The encoder comprises a series of convolutional layers designed to progressively capture the hierarchical feature representations of power towers. Each convolutional layer is typically followed by a rectified linear unit (ReLU) activation function and a max pooling operation, which serves to down-sample the spatial dimensions of the feature maps. This down-sampling strategy is instrumental in reducing the computational load and enhancing the network’s focus on the most salient features by minimising redundant spatial information.

The decoder is the element of the U-Net architecture, where the feature maps are progressively up-sampled to reconstruct the spatial resolution that are reduced during encoding. The decoder uses transposed convolutions for up-sampling and combines these larger feature maps with the attentively filtered feature maps from the encoder via skip connections. These connections are critical for restoring spatial details and context, which is lost during the encoding process, thus allowing for high-fidelity reconstruction of the segmented output.

The bottleneck functions is a pivotal point between the encoder and decoder. It processes the most abstracted form of the input data, distilling the feature maps to their most compressed and essential representations. This portion of the network typically involves a dense application of convolutional operations to enforce a robust feature encoding before the subsequent decoding phase. We have integrated into the classic U-Net architecture an attention mechanism, referred to as attention gates (AGs) Bello et al. (2019), to refine the feature extraction process, thereby enhancing the precision of segmentation in real-time applications. The integration of AGs at key junctures are positioned prior to the concatenation points of the skip connections that bridge the encoder and decoder blocks. These AGs are pivotal in executing a selection mechanism, improving the model’s capability to focus on pertinent regions within the image by enhancing salient features and diminishing the less relevant ones. This selective attention is directed by a gating signal derived from the decoder’s output, modulating the encoder’s feature response accordingly. The attention-driven modulation brought about by the AGs significantly curtails the semantic gap between the encoder’s compressed representations and the decoder’s expanded interpretations. Such a focused approach is imperative for real-time segmentation, where rapid and accurate delineation of power towers against diverse and dynamic backgrounds is crucial. The terminal part of the network is a convolutional layer with a softmax activation function that assigns to each pixel probabilities to be part of the image corresponding to the tower or to the background. This probabilistic mapping is then used to generate a segmented image, with each pixel labelled according to the class with the highest probability.

### 3.4 The computational validation

Having outlined our CNN model solution, we now turn to evaluating its “online” performance capabilities when deployed on a UAV. Our current setup includes a DJI M300 RTK UAV outfitted with a Zenmuse P1 with 3-Axis gimbal. Beyond the standard UAV configuration, we have added a custom support system that houses our onboard solution, enabling communication and control via the DJI SDK for image capture and UAV manoeuvring. The heart of this system is an NVIDIA Jetson TX2 8Gb, operating on Jetpack version 4.6.1. This setup allows us to leverage TensorRT for neural network optimisation, maximising performance given the hardware’s capabilities and the model’s computational demands. However, the ageing Jetson and its outdated Jetpack version impose limitations on the extent of TensorFlow/PyTorch optimisation, leading us to select an older CNN model for our tests. For effective real-time operational capability, we’ve determined that the model should achieve at least 7 Iterations Per Second (IPS) for basic functionality, 14IPS for moderate performance, and above 20IPS for optimal execution. Higher IPS rates enable more sophisticated filtering techniques between iterations to improve movement predictions. At 7IPS, the model is highly precise due to the absence of filtering options. At 14IPS, a two-iteration filter can be applied, and beyond 20IPS, a three-iteration Kalman filter can be utilised, accommodating models of lesser quality. This study emphasizes detection accuracy while ensuring compatibility with our hardware and the broad applicability of the system. The Attention UNet model developed and optimised via TRTEXEC (TensorRT) demonstrated 8-10IPS in a 15-min continuous feed stress test, with the Jetson operating under passive cooling at 22°C. The UAV’s flight dynamics, particularly the airflow generated by its rotors, can further enhance cooling efficiency. While the system hovers near the lower threshold of acceptable IPS for autonomous operations, not every aspects of the attention mechanism were optimal. Advances in Jetpack versions and modern embedded systems could potentially boost performance. It is noteworthy that a baseline U-Net model, lacking an attention mechanism, could reach up to 28IPS when fully optimised.

### 3.5 The training process

We have run three training processes in which an Attention U-Net model (see Section 3.3) is taught to segment images referring to power towers placed in many different background scenes (see Section 3.2). The training processes differ in the type of images in the dataset: one process is based on a dataset made of labelled physical-world images, a second one on a dataset made of synthetic images generated with the simulator (see Section 3.1), and a third one on a dataset made of a combination of physical-world and synthetic images. Each dataset is made of 1200 images. Approximately, 80% of the each dataset is used for training, with the remaining portion set aside for validation. In particular, 1020 images are used for training and 180 for validation. Note that, in the hybrid dataset, 50% of the images used for training/validation are synthetic and 50% are physical-world images.

Each training process involves several key steps. Initially, we initialise the weights of the network using a normal initialisation method. This step is crucial for facilitating effective deep network training, especially when using ReLU activation, as it helps in avoiding the vanishing gradient problem and ensures a smoother convergence. For the loss function, we used a weighted categorical cross-entropy. This approach is particularly effective in addressing class imbalances within the datasets, which is a common challenge in segmentation tasks. By weighting the classes differently, we promote a better overlap between the predicted segmentation and the actual ground truth. We employ an Adam optimiser since it is known for its efficiency in handling large datasets and complex architectures. To further enhance its effectiveness, a learning rate scheduler is integrated into the training process. This scheduler dynamically adjusted the learning rate based on the validation loss plateaus, optimising the training phase and preventing overfitting. We also implemented a hold-out validation strategy. The validation occurs after each training epoch, and is based on a composite metric including validation loss, pixel accuracy, and Intersection over Union (IoU). Validation loss is used to measure how well the network generalises to new data; pixel accuracy assessed the direct correctness of the predictions; and IoU provided a measure of precision in the segmentation task. This composite metric offers a comprehensive overview of the network capabilities, by indicating not only how accurate the network is in this segmentation task, but also how precise it is in differentiating power towers from their surroundings, and how much robust it is in segmenting images not seen during training.

## 4 Results

In this section, we first show the results of the training processes illustrated in Section 3.5, and subsequently we discuss the results of a series of post-evaluation tests run on the best trained networks. We remind the reader that our objective is to evaluate the extend to which the type of images of the dataset used for training bears upon the effectiveness of the networks in successfully distinguishing power towers from background in images taken by cameras mounted on drones engaged in inspection tasks.

**FIGURE 3:**
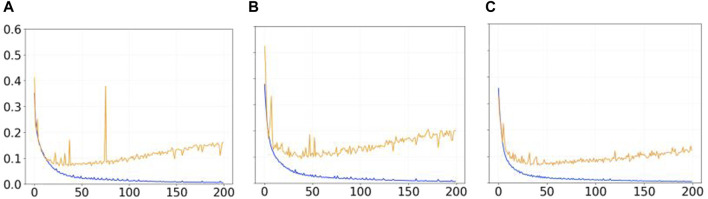
Graphs showing the trend of the Mean Error Loss for training (blue lines) and validation (orange lines) during 200 epochs with **(A)** the synthetic dataset, **(B)** the physical-world dataset, and **(C)** the hybrid dataset.

Figure 3 shows the trend of the Mean Error Loss for training and validation during 200 epochs in the three different learning processes (i.e., with synthetic images in Figure 3A, with physical-world images in Figure 3B, and with a combination of synthetic and physical-world images in Figure 3C). We notice that for all the three conditions, the initial decreasing trend for both training and validation curves is inverted after epoch 25 for the Mean Error Loss for validation (see in Figures 3A–C the orange line). This can be interpreted as a sign of overfitting, possibly due to a lack of diversity within the dataset. In order words, regardless of the types of images in the dataset, the networks, after epoch 25, seem to learn dataset-specific features rather than a effective segmentation strategy capable of performing equally well on images not used for training. We conclude that 200 epochs for training are effectively too much for this type of network which does not require that much training to reach its best performance given the size of the datasets used. The other important element from Figure 3 is that, in the condition with the hybrid dataset (see in Figure 3C), the Mean Error Loss for validation reaches lower values than in the other two conditions, suggesting that the network trained with the hybrid dataset outperforms the networks trained with the synthetic and physical-world images.

To validate this first result, we have run a series of post-evaluation tests. In particular, for each training process, we have selected two networks from the training epochs in which the Mean Errors Loss for validation is at its lowest values. This is because a low Mean Errors Loss typically correlates with higher accuracy and better generalization capabilities, thereby reducing the risk of overfitting.

Hereafter, the best two networks trained with synthetic images are referred to as *S1* and *S2*, those with physical-world images as *R1* and *R2*, and those with a combination of physical-world and synthetic images as *H1* and *H2*. These six networks have been post-evaluated with tests in which each network’s efficiency is estimated using four different metrics, and multiple types of images not seen during training. In the remaining of this Section, we describe the results of this post-evaluation analysis.

### 4.1 Multi metrics post evaluation

To compare the performances of the six different networks, we have use the following four different metrics: 1) the F1 score (hereafter, referred to as *F1*), 2) the Recall (hereafter, referred to as *Rec*), 3) the Precision (hereafter, referred to as *Pre*), and iv) the Intersection over Union (hereafter, referred to as *IoU*). Each metric is used to evaluate different aspects of the network’s performance in segmenting power towers against diverse backgrounds. In particular, *Rec* measures the proportion of actual positive cases correctly identified, indicating the network ability to capture relevant pixels. *Pre* evaluates the accuracy of positive predictions, showing how often predicted positives are correct. The *F1* harmonises recall and precision, providing a balanced measure of the network performance in cases of uneven class distribution. *IoU*assesses the overlap between predicted and actual segments, quantifying the network accuracy in segment coverage. Together, these metrics offer a comprehensive view of a network’s effectiveness in this segmentation task.

Since the segmentation of power towers images necessitates pixel-wise precision due to the intricate nature of the task, the four evaluation metrics mentioned above are grounded in a pixel-to-pixel comparison framework, in which: 1) a True Positive (TP) refers to a correct classification of a pixel identified as part of a tower; 2) a True Negative (TN) refers to a correct classification of a pixel identified as background; 3) a False Positive (FP) refers to an incorrect classification of a pixel identified as part of a tower when it is part of the background; 4) a False Negative (FN) refers to an incorrect classification of a pixel identified as part of the back ground when it is part of a tower. We have chosen to post-evaluate each network on the three different types of dataset: the synthetic dataset (hereafter, referred to as *S* dataset), the real-world images dataset (hereafter, referred to as *R* dataset), and the hybrid dataset (hereafter, referred to as *H* dataset). Each dataset is made of 450 images not seen during training. For the *H* dataset, 225 are physical-world and 225 synthetic. The composition of these datasets ensures a thorough and effective evaluation of the networks’ performance across different conditions, which is crucial for the precise segmentation of power towers.

**FIGURE 4:**
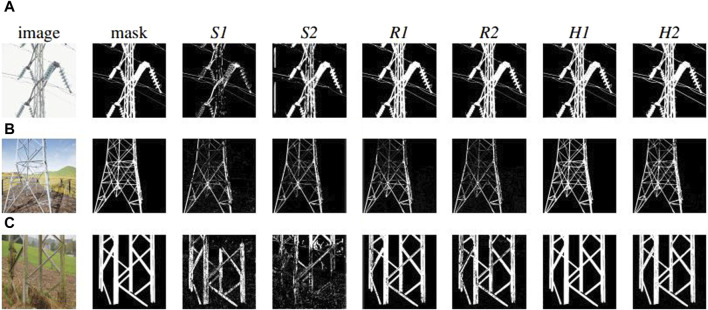
Examples of the results at the post-evaluation tests by the six selected best networks *S1*, *S2*, *R1*, *R2*, *H1*, and *H2* on: **(A)** a physical-world image captured with a positive pitch, featuring a power tower against a clear sky background; **(B)** a synthetic image on a horizontal pitch, depicting a power tower in a field, surrounded by a metal fence; **(C)** a physical-world image with a negative pitch, showcasing a grass and dirt foreground with the sky visible at the top.

The results, shown in Table 2, indicate that, the best performances in all the metrics are those obtained by the networks trained with the hybrid dataset (see Table 2 row *H1* and *H2* for all metrics). These best performances are recorded with either synthetic images (see Table 2, column *S*, for *Rec* and *Pre*) or with the *H* dataset (see Table 2, column *H*, for *F1* and *IoU*). This result suggests that the networks *H1* and *H2* possess a remarkable ability to effectively interpret both synthetic and physical-world images, with a strong generalisation capabilities, particularly in discerning the shape of power towers across varying environmental conditions (Figure 4).

**TABLE 2:** Table showing the performances of the six networks (*S1*, *S2*, *R1*, *R2*, *H1*, *H2*) at the post-evaluation analysis. Each network is post-evaluated on the three different types of datasets (i.e., the *S*, the *R*, and the *H*), and the performances are shown with respect to four metrics (i.e., the *F1*, the *Rec*, the *Pre*, and the *IoU*). The best performance for each metric is indicated in bold.

	*F1*			*Rec*			*Pre*			*IoU*		
	*S*	*R*	*H*	*S*	*R*	*H*	*S*	*R*	*H*	*S*	*R*	*H*
*S1*	0.834	0.855	0.847	0.824	0.909	0.876	0.864	0.815	0.834	0.729	0.764	0.751
*S2*	0.819	0.866	0.848	0.806	0.920	0.875	0.860	0.826	0.840	0.713	0.780	0.754
*R1*	0.953	0.946	0.969	0.957	0.949	0.969	0.952	0.947	0.971	0.890	0.947	0.971
*R2*	0.961	0.950	0.975	0.977	0.955	0.982	0.948	0.947	0.969	0.913	0.947	0.969
*H1*	0.992	0.955	0.986	0.994	0.961	0.988	**0.992**	0.950	0.984	0.978	0.950	**0.984**
*H2*	0.993	0.957	**0.987**	**0.996**	0.964	0.992	0.990	0.951	0.983	0.980	0.951	0.983

These post-evaluation tests have also given other more interesting and unequivocal indications. First of all, the data shown in Table 2 tells us that the networks trained with hybrid images (i.e., *H1*and *H2*), perform, for all metrics, slightly better than the best networks trained with physical-world images when evaluated with the *R* dataset (see Table 2, row *H1* and *H2*, and *R1* and *R2*, for all columns *R*). This indicates that networks *H1 H2* are highly effective in accurately identifying, in physical-world images, (see Figures 4A, C) positive cases (high precision), by effectively capturing a majority of the TP instances (high recall). Moreover, networks *H1* and *H2* prove effective in maintaining a balanced trade-off between precision and recall (reflected in the F1 score), and in accurately delineating the spatial boundaries of the objects (indicated by IoU). Note also that, both the performances of networks *R1* and *R2* as well as of networks *H1* and *H2* are very close to the maximum scores (i.e., 1) that a network can get in each of these four metrics. The consistent high scores in all metrics is a sign of networks that have achieved a good balance between underfitting and overfitting, or in other words, an effective trade-off between bias and variance. Thus, they promise to be highly reliable to be effectively deployed in a physical world power tower inspection process.

Another important element from Table 2 concerns the scores at metric *Rec* for networks *H1* and *H2* and networks *R1* and *R2*. The high score at *Rec* by these networks indicates that these networks are particularly effective at identifying most of the relevant instances of power towers, demonstrating high sensitivity in detecting the objects of interest. However, it is worth noticing that for both *H1* and *H2* and *R1* and *R2*, the scores at *F1* and *Pre* are lower in comparison to those at *Rec*. This points to the fact that these networks prioritise detecting as many true positives as possible (high *Rec*) but at the cost of including more false positives (lower *Pre*). The metric *Pre* assesses how many of the predicted positives are actually true positives. Therefore, a lower score at *Pre* than at *Rec* implies that while these networks detect most of the power towers, not all pixels considered part of a tower are correctly classified.

The other clear result from the data in Table 2 refers to the performances of networks trained with only synthetic images. These networks (i.e., *S1* and *S2*) obtained the lowest performances in all tests. *S1* and *S2* record the worst performances even with respect to images of the same type of those experienced during training (see Table 2, rows *S1* and *S2*, for all columns *S*). This suggests that the limited variety of photogrammetry-based power towers in synthetic images leads to overfitting, which occurs despite the high variability in the background characterising the synthetic images used for training. Therefore, we conclude that variability during training with respect to the features of the power towers, instead of the background, is crucial for achieving good generalization. The relatively high score at the metric *Rec* for *S1* and *S2* indicates that these networks are effective at identifying most of the relevant instances of power towers in all datasets (see Table 2, rows *S1* and *S2*, for column *S*, metric *Rec*). Essentially, this suggests that these networks are capable of detecting the presence of power towers with a high degree of sensitivity. However, the low *IoU* score implies that while these networks are successful in identifying power towers, their precision in outlining or segmenting the towers is inadequate (see Table 2, rows *S1* and *S2*, for column *S*, metric *IoU*). This combination of high *Rec* and low *IoU* scores typically points to networks that show over-detection; that is, they correctly identify the objects of interest but also inaccurately include a significant amount of the surrounding area in their predictions. This characteristic is particularly critical in applications where accurate delineation of the object is as important as its detection.

As far as it concerns networks *S1* and *S2*, it is worth noticing that although their capabilities are somewhat limited and not as robust as those of the other types of networks, they still manage to perform relatively well (see Figure 4B). This is thanks to a synthetic dataset in which images are generated by using substantial noise filtering techniques and the application of a Kalman filter Kalman (1960). These techniques are particularly crucial to boost the performance of networks that, otherwise tend to exhibit lower scores at *IoU* metric, indicating a tendency to inaccurately include surrounding areas in their predictions. By employing noise filtering and the Kalman filter, the risk of such inaccuracies can be significantly reduced, enhancing the practical usability of the networks trained with the *S* dataset despite their inherent limitations in comparison to models trained with the *R* and *H* dataset.

Finally, a significant aspect of our findings pertains to the influence of synthetic images on the training of networks *H1* and *H2*. In particular, this type of images does not adversely affect the networks’ ability to accurately interpret and analyse physical-world images. This observation is particularly noteworthy as it highlights the efficacy of the photogrammetry techniques and the simulation tool used to create the synthetic images used for training. We can claim that the simulation tool we employed generates photorealistic images of power towers, sufficiently detailed to effectively contribute to shape the segmentation mechanisms underlying the good performances of networks *H1* and *H2*. The simulated images, therefore, not only augment the diversity of the training dataset but also contribute positively to the networks’ ability to generalise and to perform effectively across different scenarios. This finding has significant implications for the use of simulated data in training robust networks, especially in contexts where physical-world data might be limited or too challenging to obtain.

### 4.2 Comparison with mask2former

The U-Net architecture described in Section 3.3 has been chosen by taking into account a important trade-off between performance and computational resources which is imposed by the technology currently available to run the power tower inspection task by the company that financially supports this study. Generally speaking, as far as it concerns the domain of CNNs, the performance and the required computational resources to run the network tend to be positively correlated, meaning that a progressively better performance is likely to require networks that are more costly in terms of computational resources.

In this Section, we show the results of a comparative test that quantifies the performance gain obtained by replacing the U-Net with a computationally more costly state-of-the-art CNN for the image segmentation task described in this study. In particular, we train a mask2former model (m2f) Cheng et al. (2022), integrated with a Swin-B backbone Liu et al. (2022), on the same datasets (*S*, *R*, *H*) used for training the Att-U-Net model. Just like the U-Net model, the Mask2Former model gets the same size of input and output (512 width and 512 height image). The comparative results, illustrated in Table 3, indicate a slightly superior performance of the mask2former model over the U-Net variant. Consistent with earlier findings, the Mask2Former trained with the *H* dataset exhibits an overall enhanced performance. Nevertheless, contrary to what observed with the U-net, the Mask2Former trained with the other two datasets (i.e., *S*, *R*), yields results that are comparably close to the mask2former Hybrid model trained with the *H* dataset. The results of this test confirm that larger computational resources make possible to run more effective networks which produce better performances. However, the U-Net remains an effective solution, that guarantees an excellent performance at reduced computational costs compared to other slightly more effective networks.

**TABLE 3:** Table comparing the performance of the U-Nets (S2, R2, and H2) and of the mask2former (m2f) across different training regimes, using the F1 score (*F1*), Recall (*Rec*), Precision (*Pre*), and IoU (*IoU*) as evaluation metrics. See also the caption of Table 2 for more details. In each column, the highest value is highlighted to clearly identify the top-performing model for every metric and data type.

	*F1*			*Rec*			*Pre*			*IoU*		
	*S*	*R*	*H*	*S*	*R*	*H*	*S*	*R*	*H*	*S*	*R*	*H*
m2f S	**0.995**	0.977	0.986	**0.996**	0.985	0.990	**0.995**	0.969	0.982	**0.990**	0.955	0.973
*S2*	0.819	0.866	0.848	0.806	0.920	0.875	0.860	0.826	0.840	0.713	0.780	0.754
m2f R	0.989	**0.992**	0.992	0.991	0.992	0.992	0.987	**0.992**	**0.992**	0.979	**0.984**	0.985
*R2*	0.961	0.950	0.975	0.977	0.955	0.982	0.948	0.947	0.969	0.913	0.947	0.969
m2f H	0.993	**0.992**	**0.993**	0.995	**0.993**	**0.994**	0.992	0.991	**0.992**	0.987	**0.984**	**0.986**
*H2*	0.993	0.957	0.987	**0.996**	0.964	0.992	0.990	0.951	0.983	0.980	0.951	0.983

## 5 Discussion and future works

In this Section, we discuss how the Convolution Neural Network illustrated in Section 3.3 could be integrated into a larger control system to automate the behaviour of UAVs engaged in power tower inspection tasks, while being trained with a hybrid dataset explained in Section 3.2.

Since after segmentation, the pixels representing the power tower are labelled differently from those referring to other objects or background, a relatively simple algorithm can reliably extract the position of the power tower with respect to the camera and consequently with respect to the UAV. This information is subsequently used to plan UAVs movements that avoid collisions and generate those perspectives on the tower that are instrumental for the inspection task.

We believe that, by drawing inspiration from basic lane assistance technology in automobiles, an advanced assisted flight control system can be developed to monitor the precision of images captured during flight in real time. By integrating a CNN into the loop between the pilot and the Unmanned Aerial Vehicle (UAV), a PID (Proportional, Integral, Derivative) controller can be implemented to manage the UAV’s roll axis. By processing the edge position of power towers in the images, the system can accurately determine the UAV’s positional deviation relative to the tower. This means the pilot can concentrate on altitude adjustment and maintaining a safe distance, while the AI system ensures the power tower is centrally aligned in the captured images, guaranteeing comprehensive coverage of the tower.

The task of centring the power tower on the camera image while maintaining an appropriate distance from the tower can be efficiently managed by the camera system itself, provided certain parameters of the power tower, such as the width of its main structure, are known (see also Maître et al., 2022, as an example). With this approach, the UAV is free from additional, bulkier equipment, such as proximity sensors like LiDAR. Utilizing the known width of the tower and the expected pixel coverage in the image, the actual distance from the power tower can be deduced by applying the Ground Sampling Distance (GSD) algorithm. The GSD, which represents the distance between two consecutive pixel centres measured on the ground, can be calculated in the following:

$$GSD=\frac{H\times S}{F\times R};$$ (1)

to

$$\alpha =\frac{GSD}{R}=\frac{H\times S}{F};$$ (2)

where *α* is the optimal pixel size, and GSD, expressed in meters, represents the distance between two consecutive pixel centers measured on the ground. GSD measures are directly influenced by several factors including the altitude (*H*) of the UAV above the ground, which is also measured in meters. The size of the sensor’s pixel (*S*), often provided by the manufacturer or calculable from the sensor size and resolution, plays a crucial role as well, alongside the focal length (*F*) of the sensor’s lens, which is measured in meters. Additionally, the resolution of the image (*R*), defined as the number of pixels along one dimension of the image, is a critical parameter in determining the GSD.

By knowing the width of the power tower and measuring the number of pixels it occupies in images taken from an optimum distance, an approximate size per pixel at each inference can be computed in the following:

P=W/R; (3)

where the pixel size (*P*) is measured in centimetres and represents the dimensions of an individual pixel. The width of the power tower’s main structure is denoted as *W*. Additionally, the resolution of the image (*R*) is defined by the number of pixels along one dimension of the image, indicating the total pixel count in that direction.

$$\text{Movement}=\left\{\begin{array}{cc} \text{Move Closer}\; & \text{if }P>\alpha \\ \text{Stay}\; & \text{if }P=\alpha \\ \text{Move Away}\; & \text{if }P<\alpha \end{array}\right.$$

Finally, reinforcement learning could be employed to interpret the mask as input for generating potential UAV movements. Given that RGB images are information-dense, with 3 × 8 bits allocated for each pixel, the mask’s simplified binary [0, 1] input array could significantly streamline the image data. This simplification aids in developing a solution capable of self-centring, by learning from a more condensed input set. Consequently, this approach could lead to the optimisation of a smaller neural network, enhancing efficiency.

In terms of future work, we aim to evaluate the efficacy of different control options. Our comparative study will include audits of several systems: 1) manual control by a human pilot; 2) a flight assistant using a multisegment LiDAR; 3) an advanced autopilot that combines GPS-based online path planning with LiDAR sensors; 4) a computer vision-based approach augmented with GPS for high-precision positioning; and 5) a fully autonomous system powered by computer vision. We will assess these systems based on the speed of data acquisition, the accuracy and consistency of pylon centring in the images, and their adaptability to challenging environments. This comparative analysis will be facilitated by utilising professional test power tower and its digital twin, which were integrated into our simulation for this study and employed in the training of our model.

## 6 Conclusion

We have illustrated the results of a set of simulations in which we trained a type of CNNs, referred to as U-Net, to identify power towers from background in images depicting a variety of scenarios. In particular, we compared the performances of U-Nets trained with three different types of datasets: 1) a dataset made of synthetic images; 2) a dataset made of physical-world images; and 3) a hybrid dataset mixing synthetic and physical-world images. Post-evaluation tests have shown that the U-Nets trained with the hybrid datasets outperform the other U-Nets in all the four evaluations metrics used to estimate the precision with which the networks segment physical-world and synthetic images in order to distinguish powers towers from the background.

From these results, we draw the following conclusions. First of all, the synthetic images generated by our simulator effectively contribute to improve the performances of U-Nets engaged in the power tower segmentation task. Given the complexity, high risks, and the high costs generally associated to power tower inspection with traditional methods (see Section 1 for details), our research work contributes to reduce costs and risks by delivering computer-vision algorithms that facilitate the engineering of autonomous UAVs capable of replacing humans in the inspection task. In particular, the innovative value of our work is in the development of a methodology that generates high performing state-of-the-art image-segmentation algorithms (i.e., the U-Net) by minimising the needs of physical-world images used for training them. A sufficiently large dataset of physical-world images in the context of power tower inspection by UAVs can be difficult to acquire. Moreover, once acquired, the inevitable image-annotation process can be extremely costly and time-consuming. The methodology illustrated in this research work demonstrates that, it is possible to largely reduce the quantity of physical-world images, and consequently the costs and the time of their acquisition and annotation, by relying on a particular type of synthetic images. These synthetic images are characterised by being generated with a simulator the can facilitate the rendering of multiple different scenes with a particular care of details, and by photogrammetry used to build high-precision models of different types of powers towers (see Section 3.1 for details).

A further contribution of our work, can be identified in the multiple functionalities of the simulator that can be used not only to generate synthetic images, but also to test and to evaluate the effectiveness of different control algorithms in guiding autonomous UAVs in performing the different operations (e.g., navigation, obstacle avoidance, identification of problems, etc.) related to the power tower inspection task. This can be done thanks to the integration into the simulator of libraries such as the physics engine Unreal, required to simulate the UAVs flight dynamics, as well as the dynamics of collisions. In this study, we have limited ourselves to test the best trained U-Nets on the image-segmentation task. However, in the future, we will tests and validate our algorithms on simulated UAVs required to fly and to inspect power towers in a variety of scenarios as those described in Section 3.2. This will require the integration of U-Nets trained for image-segmentation task into a more complex UAV control system that takes care of all the other processes needed to make the vehicle fully autonomous in the inspection task. We are also planning to test and to validate our algorithms on physical UAVs, such as those owned by one of funding body of this work, the company Qualitics (see https://www.qualitics.ai/) that operates in the domain of power tower inspections with UAVs in Belgium. These tests with physical UAVs will be important not only to directly evaluate the capability of the control system, designed in simulation, to deal with the complexity of the physical world, but also to indirectly evaluate the effectiveness of the methodological toolkit we developed and used to design the components of this controller.

## Data Availability

The data analyzed in this study is subject to the following licenses/restrictions: the dataset used in this study is created and owned by Qualitics. The use of this dataset is restricted to them. Requests to access these datasets should be directed to Guillaume Maitre, guillaume.maitre@qualitics.ai.
